# Targeting UCHL3 attenuates pathological markers in neuronal models of Huntington’s disease

**DOI:** 10.1093/brain/awag028

**Published:** 2026-01-24

**Authors:** Hasan Ishtayeh, Elena Battistoni, Sharon Pochtar, Tyne L M McHugh, Kizito-Tshitoko Tshilenge, Brian Rossmiller, Fatima Amer-Sarsour, Yevgeny Berdichevsky, Noam Muchtar, Miguel Weil, Lisa M Ellerby, Avraham Ashkenazi

**Affiliations:** Department of Cellular, Developmental, and Regenerative Biology, Gray School of Medical Sciences, Gray Faculty of Medical and Health Sciences, Tel Aviv University, Tel Aviv 6997801, Israel; Buck Institute for Research on Aging, Novato, CA 94945, USA; Department of Cellular, Developmental, and Regenerative Biology, Gray School of Medical Sciences, Gray Faculty of Medical and Health Sciences, Tel Aviv University, Tel Aviv 6997801, Israel; Sagol School of Neuroscience, Tel Aviv University, Tel Aviv 6997801, Israel; Buck Institute for Research on Aging, Novato, CA 94945, USA; Leonard Davis School of Gerontology, University of Southern California, Los Angeles, CA 90089, USA; Buck Institute for Research on Aging, Novato, CA 94945, USA; Buck Institute for Research on Aging, Novato, CA 94945, USA; Department of Cellular, Developmental, and Regenerative Biology, Gray School of Medical Sciences, Gray Faculty of Medical and Health Sciences, Tel Aviv University, Tel Aviv 6997801, Israel; Department of Cellular, Developmental, and Regenerative Biology, Gray School of Medical Sciences, Gray Faculty of Medical and Health Sciences, Tel Aviv University, Tel Aviv 6997801, Israel; The Shmunis School of Biomedicine and Cancer Research, The George S. Wise Faculty for Life Sciences, Tel Aviv 6997801, Israel; Sagol School of Neuroscience, Tel Aviv University, Tel Aviv 6997801, Israel; The Shmunis School of Biomedicine and Cancer Research, The George S. Wise Faculty for Life Sciences, Tel Aviv 6997801, Israel; Buck Institute for Research on Aging, Novato, CA 94945, USA; Leonard Davis School of Gerontology, University of Southern California, Los Angeles, CA 90089, USA; Department of Cellular, Developmental, and Regenerative Biology, Gray School of Medical Sciences, Gray Faculty of Medical and Health Sciences, Tel Aviv University, Tel Aviv 6997801, Israel; Sagol School of Neuroscience, Tel Aviv University, Tel Aviv 6997801, Israel

**Keywords:** Huntington’s disease, aggregate-prone proteins, autophagy, induced pluripotent stem cells

## Abstract

Huntington’s disease is an autosomal dominant neurodegenerative disease with a well-characterized genetic aetiology of a CAG expansion mutation in the huntingtin (*HTT*) gene, yet it remains without a cure. The hallmark of Huntington’s disease is the accumulation of intraneuronal aggregates of mutant HTT protein and polyglutamine (polyQ)-containing fragments, which causes impaired proteostasis and is an important Huntington’s disease therapeutic target. Aggregate-prone protein clearance primarily occurs through the autophagy-lysosome pathway and the ubiquitin-proteasome system, both of which can be modulated by deubiquitinating enzymes (DUBs).

This study investigates the role of the DUB ubiquitin C-terminal hydrolase L3 (UCHL3) in modulating polyQ-mediated aggregation and toxicity. UCHL3 has previously been identified as a potential therapeutic target in cancer. We used Huntington’s disease models, including primary mouse neurons, patient fibroblasts and patient-derived medium spiny neurons, which are the most vulnerable to HTT polyQ toxicity.

Genetic lowering of UCHL3 decreased polyQ aggregates and increased autophagosome-lysosome fusion events. This was accompanied by STAT3 induction, which protects against neuronal proteotoxic stress. Furthermore, treatment with a small-molecule inhibitor of UCHL3 recapitulated the effects of UCHL3 lowering and attenuated pathological markers in Huntington’s disease medium spiny neurons.

These results provide a foundation for further exploration of UCHL3 inhibitors in the context of Huntington’s disease and underscore the biological connection between cancer and neurodegeneration for drug repurposing strategies.

## Teaser

Ishtayeh *et al.* identify the cancer-associated deubiquitinating enzyme UCHL3 as a potential therapeutic target in Huntington’s disease. Genetic and pharmacological inhibition of UCHL3 in mouse and patient-derived neurons reduced mutant huntingtin aggregation and toxicity by enhancing protective proteostasis stress responses.

## Introduction

Patients with Huntington’s disease (HD) exhibit involuntary movements, emotional disturbances, and cognitive decline during the progression of the disease.^1^ The cause of HD is a CAG trinucleotide repeat expansion mutation in exon 1 of the *HTT* gene. This mutation causes polyglutamine (polyQ) expansion within the HTT N-terminus, and age-at-onset of the disease decreases with increasing CAG length.^2^ HD is associated with progressive degeneration of neurons in the cortex and striatum, with the gamma-aminobutyric acid (GABA)ergic medium spiny neurons (MSN) being the most vulnerable to the polyQ expansion mutation.^3,4^ In addition to HD, at least eight different neurodegenerative diseases are caused by expansion mutations of the polyQ domain in different proteins.^5^ These diseases share the pathological features of selective neuronal loss associated with the aggregation of the polyQ-expanded proteins forming intraneuronal inclusions.^5^ It is clear that polyQ domain expansions drive the pathology in these diseases because both isolated polyQ expansions and mutant HTT exon-1 fragments are toxic.^6^ Our findings, consistent with those of other groups, suggest that autophagy impairment is one of the mechanisms underlying the pathogenesis of the polyQ expansion mutation.^7,8^ Autophagy, a cellular self-digestion process, is a lysosome-dependent, evolutionarily conserved degradation pathway for clearance of aggregated proteins and damaged organelles.^9,10^ Induction of autophagy decreases aggregate-prone mutant HTT levels, and alleviates its toxicity in cell and mouse models of HD.^11^

In the search for new druggable modulators of polyQ aggregation and toxicity, we examined emerging therapeutic cancer targets implicated in protein degradation. Ubiquitin conjugation to target proteins plays a pivotal role in regulating their degradation via autophagy or the proteasome.^12^ Ubiquitination is a highly dynamic and transient process, partly because deubiquitinating enzymes (DUBs) effectively remove ubiquitin tags of mono- and polyubiquitination. DUBs have an important role in protein homeostasis (proteostasis) and regulating signalling pathways in neurons.^12^ In fact, growing evidence from both human and mouse studies indicates that mutations in certain DUBs can result in neurological disorders.^7,13^

The DUB UCHL3 is a promising therapeutic target for certain cancers as it plays an important role in tumorigenesis, but its functions in nervous system pathologies are still unclear. The expression of UCHL3 is elevated in tissues of non-small cell lung cancer, breast cancer and ovarian cancer.^14,15^ UCHL3 was also found to be involved in promoting tumour growth, migration and invasion in several cancer types.^16-18^ To determine if UCHL3 could serve as a target for future therapeutic interventions, small molecule inhibitors of UCHL3 have been developed for cancer research.^19,20^ These molecules have not yet demonstrated protective effects in animal models, possibly because of instability *in vivo,* but their development for preclinical and clinical studies is ongoing. One of the most studied UCHL3 inhibitors is TCID, also known as 4,5,6,7-tetrachloroindene-1,3-dione; it is a cell-penetrating small molecule inhibitor with high potency and selectivity.^20^ For example, TCID treatment was shown to prevent cell proliferation and colony formation in non-small-cell lung cancer and decreased the growth and invasion of hepatocellular carcinoma cells.^16,18,19^

In this study, we used genetic and pharmacological approaches to elucidate the functions of UCHL3 in regulating neuronal polyQ-mediated aggregation and toxicity. We revealed that neuronal depletion of UCHL3 reduced polyQ aggregation and promoted autophagy involving increased transcriptional levels of signal transducer and activator of transcription 3 (STAT3). To model the pathogenic events in primary human neurons affected by the polyQ-expansion mutation, we investigated MSN from the isogenic HD patient-derived induced pluripotent stem cells (iPSCs) we developed.^21,22^ The corrected iPSCs preserved the characteristics of pluripotent stem cells, and the differences between the lines were attributed to mutant HTT-specific effects. UCHL3 inhibition in HD patient-derived MSN ameliorated nuclear aggregation and increased MSN levels of DARPP-32, highlighting UCHL3 as a promising potential target in HD.

## Materials and methods

Detailed materials and methods are provided in the Supplementary material, ‘Methods’ section and Supplementary Table 1.

### Mouse and human neurons

Cortical neurons were cultured from wild-type C57BL/6J mouse embryos at embryonic Day 17 (E17) as we have previously described.^23^ C116 and HD72 neural NSC were generated as previously described^22^ and then differentiated into MSN according to our established protocol.^22^

### Immunohistochemistry of human control and HD brain

Formalin-fixed, paraffin-embedded cortical sections from HD patients and controls from the Harvard Brain Bank were deparaffinized, rehydrated, and subjected to antigen retrieval in Tris-EDTA buffer (pH 9.0) in a crockpot for 20 min, followed by two washes in PBS for 10 min. Slides were blocked in 5% normal donkey serum with 0.5% Triton X-100 for 1 h at room temperature, then incubated overnight at 4°C with mouse anti-HTT (MAB5490, 1:100) and rabbit anti-UCHL3 (1:100) antibodies. After three 10-min PBS washes, sections were incubated with Alexa Fluor-conjugated secondary antibodies (1:500) for 1 h at room temperature. After PBS washes, slides were incubated with DAPI and TrueBlack Lipofuscin Autofluorescence Quencher before mounting with ProLong Gold Antifade Mountant and glass coverslips. Confocal images were acquired on a Zeiss LSM 980 microscope. Manders’ co-localization coefficients (tM2) representing the fraction of HTT signal overlapping with UCHL3 were calculated using the Coloc2 plugin in ImageJ after Costes automatic thresholding and background subtraction.

### Analysis of UCHL3 transcript levels in HD patient cortex

RNA sequencing data for post-mortem human dorsolateral prefrontal cortex from HD patients were obtained from the publicly available dataset GSE64810. Normalized transcript per million (TPM) values for UCHL3 were extracted for HD cases with corresponding HTT CAG repeat lengths. Pearson correlation analysis between UCHL3 TPM and CAG length was performed using GraphPad Prism v10.3.1.

## Results

### Neuronal UCHL3 regulates polyglutamine expansions and autophagy

**Figure 1:**
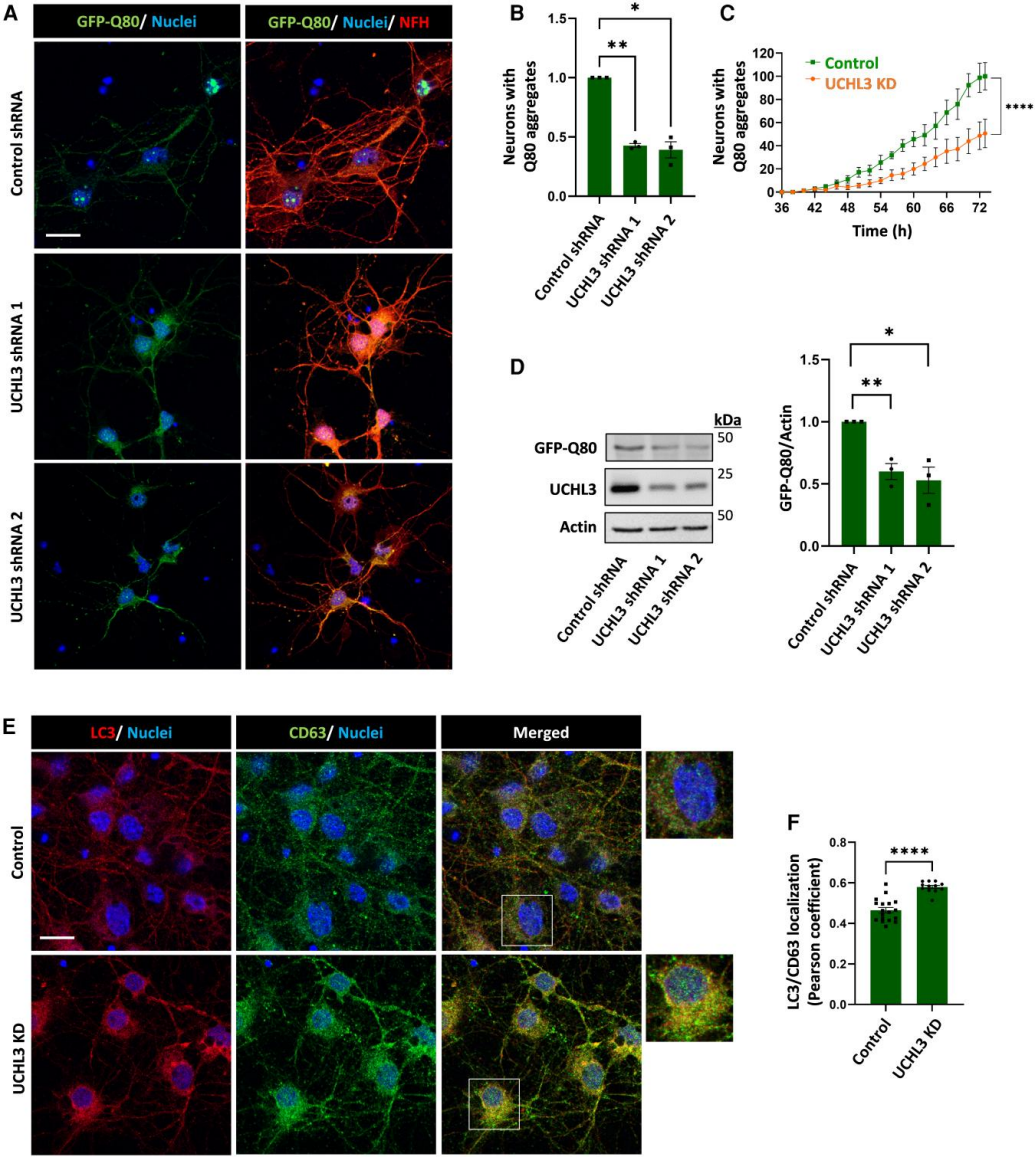
**Genetic lowering of UCHL3 in primary neurons reduces aggregation of polyQ expansions and induces autophagosome-lysosome fusion**. (**A–C**) Mouse primary cortical neurons were transduced with lentiviruses encoding for scrambled control shRNA and distinct UCHL3 shRNAs. The neurons were co-transduced with lentiviruses encoding for GFP-Q80. (**A**) Representative images include the neuronal morphological marker NFH (red). Scale bar = 20 μm. (**B**) Quantification of neurons with nuclear GFP-Q80 aggregates in *n* = 3 independent cultures. *n* > 100 neurons counted per condition per culture. Results represent means ± SEM normalized to control. (**C**) Time-lapse imaging of GFP-Q80 aggregate formation in control (scrambled shRNA) and UCHL3 KD (UCHL3 shRNA) neurons. At least 70 000 cell bodies were sampled for the analysis. Results represent means ± SEM fraction of neurons with aggregates. *t* = 0 is defined as the viral transduction time. (**D**) GFP-Q80 protein levels in UCHL3 KD neurons normalized to control neurons. *n* = 3 independent cultures. (**E** and **F**) Quantification of co-localization of the autophagy marker LC3 (red) and late endosome/lysosome marker CD63 (green) in the control and UCHL3 KD neurons. Pearson correlation coefficient was analysed in different imaging fields from *n* = 3 independent cultures. Representative images are shown (scale bar = 10 μm). (**B** and **D**) *P-*values for differences between means were assessed using a one-way ANOVA and *post hoc* Dunnet test. (**C**) Two-way ANOVA and *post hoc* Sidak’s test. (**E** and **F**) Student’s *t*-test. *P < 0.05, **P < 0.01, ****P < 0.0001. KD = knockdown; SEM = standard error of the mean.

To elucidate the role of UCHL3 in expanded polyQ pathology, we analysed the expression levels of UCHL3 in the prefrontal cortex of HD patients with varying CAG repeat expansion mutations. UCHL3 levels were directly correlated with CAG repeat size (Supplementary Fig. 1A) and UCHL3 colocalized with HTT in post-mortem HD brain tissues (Supplementary Fig. 1B and C). Next, we isolated primary neurons from the cortex of mouse embryos and transduced them with a lentiviral vector encoding an isolated stretch of 80 glutamine residues tagged with GFP (GFP-Q80). Five days post-transduction, intranuclear aggregation of the GFP-Q80 was detected in neurons by fluorescent imaging (Fig. 1A). This phenomenon of intranuclear aggregation has also been detected in the brains of HD patients and mouse models of the disease by staining for HTT or the polyQ.^3^ Using lentiviral vectors expressing shRNA targeting UCHL3, we were able to knock down (KD) endogenous UCHL3 in the GFP-Q80-expressing neurons. We used two different UCHL3 shRNAs together with a scramble control shRNA and analysed the percentage of neurons with aggregates in the different conditions by counting *n* = 250–350 nuclei with GFP-Q80 in independent neuronal cultures (Fig. 1A). The decrease in UCHL3 levels by both shRNAs showed significant reduction in the number of neurons with established Q80 aggregates (Fig. 1B). Automated time-lapse imaging was used to track aggregate formation. In these experiments, we found that UCHL3 depletion also prevented initial Q80 aggregation in the neurons (Fig. 1C and Supplementary Videos 1 and 2). We next assessed the neuronal lysates for the total levels of GFP-Q80 and found that GFP-Q80 levels were decreased following UCHL3 KD (Fig. 1D).

Proteins with aggregate-prone polyQ expansions are substrates of autophagy, and when autophagy is induced, the levels and aggregation of expanded polyQ proteins are decreased due to their degradation in lysosomes.^11^ We therefore examined the regulation of this process by UCHL3 in the primary neurons. To assess autophagosome-lysosome fusion in control and UCHL3-KD neurons, we quantified LC3 (autophagosome marker) and CD63 (lysosome/late endosome marker) double-labelled vesicles (Fig. 1E). UCHL3 KD increased the colocalization of endogenous LC3 with CD63, suggesting that autophagosome-lysosome fusion is enhanced (Fig. 1F), further supporting the notion that UCHL3 negatively regulates the autophagy-lysosome degradation pathway.

### UCHL3–STAT3 regulatory pathway

**Figure 2:**
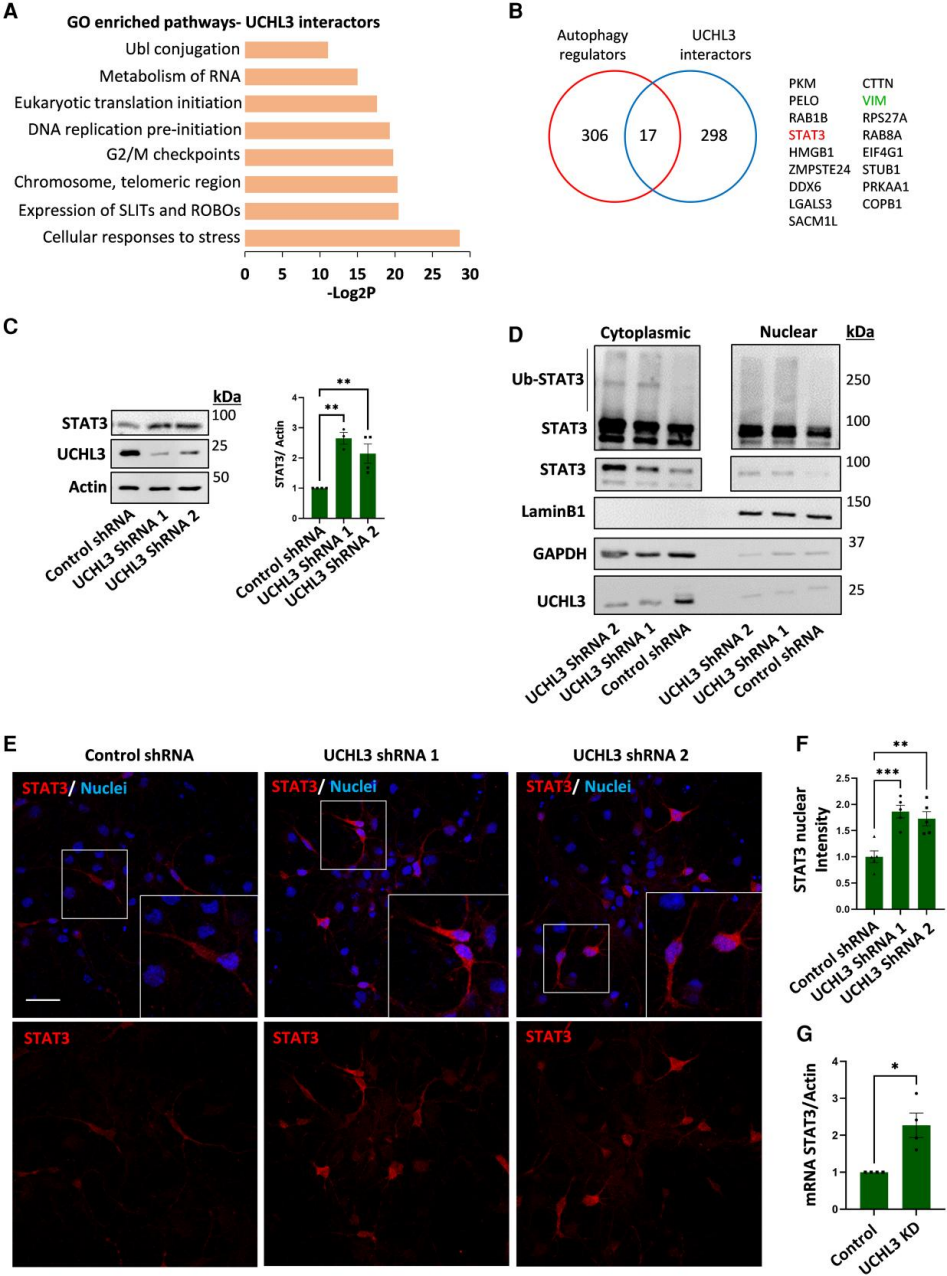
**UCHL3 regulates STAT3 levels and nuclear localization.** (**A**) Cells were transfected with HA-UCHL3 vector or empty vector and UCHL3 was immunoprecipitated from cell lysates with HA antibodies. The immunocomplexes were analysed for UCHL3 interactors by proteomic analysis. Functional pathway analysis of the identified interactors was performed by DAVID. (**B**) Selected autophagy-regulating hits was performed by crossing the interactors with the UniProt autophagy database. (**C**) STAT3 protein levels were quantified in the control and UCHL3 KD neurons in *n* = 3–4 independent cultures. Results represent means ± SEM normalized to control. (**D**) Nuclear and cytoplasmic fractions of control and UCHL3 KD neurons analysed for ubiquitinated and non-ubiquitinated STAT3 levels; as well as the levels of GAPDH as a cytoplasmic marker and LaminB1 as a nuclear marker. (**E** and **F**) Quantification of average intensity of nuclear STAT3 in control and UCHL3 KD neurons. Results are representative of *n* = 3 independent cultures. Image scale bar = 50μm. (**G**) RT-qPCR measuring *STAT3* mRNA levels normalized to actin mRNA in control and UCHL3 KD neurons. *n* = 4 replicates from two independent cultures. (**C**, **E** and **F**) One-way ANOVA and *post hoc* Dunnet test. (**G**) Student’s *t*-test. *P < 0.05, **P < 0.01, ***P < 0.001. KD = knockdown; SEM = standard error of the mean.

UCHL3 is a cysteine protease with deubiquitination activity cleaving polyubiquitin chains from target proteins. We employed liquid chromatography-tandem mass spectrometry (LC-MS/MS) to identify 305 interactors with UCHL3 in pulldown experiments. Functional enrichment analysis of the protein clusters revealed UCHL3-regulated pathways involved in cellular response to stress, cell proliferation (chromosome, telomeric region, G2/M checkpoints, DNA replication pre-initiation), translation initiation, RNA metabolism and ubiquitin-like protein conjugation, among others (Fig. 2A and Supplementary Table 2). It has been shown that UCHL3 overexpression can impact the levels of proteins involved in cell cycle and mitosis implicated in cancer development and progression, which is in line with our findings.^24^ Moreover, vimentin, a known UCHL3 target associated with hepatocellular carcinoma,^16^ was identified in the proteomics, further validating our experimental setting (Fig. 2B).

To gain further insights for neurons, which are post-mitotic cells, we performed an additional analysis comparing known autophagy proteins from UniProt database to the proteins identified by our LC-MS/MS analysis. The Venn diagram in Fig. 2B presents a subset of shared proteins, among them is STAT3, which protects HD patient-derived neurons from cell death.^8^ UCHL3 KD neurons showed a significant increase in STAT3 levels and its subsequent nuclear localization compared to control scrambled shRNA-expressing neurons (Fig. 2C, E and F). We performed a biochemical separation of cytoplasmic and nuclear fractions to assess whether UCHL3 affected the ubiquitination state of STAT3 in the different neuronal fractions. In agreement with the nuclear localization experiment, our biochemical analysis also showed increased nuclear STAT3 levels in UCHL3 KD neurons without alteration in STAT3 polyubiquitination (Fig. 2D). This suggests that UCHL3 may regulate STAT3 via alternative mechanisms. Indeed, one such mechanism was the transcriptional regulation of *STAT3* mRNA levels (Fig. 2G). Overall, our results thus far support a model where UCHL3 suppression protects from neuronal stress of polyQ aggregation and activates protective pathways highlighted by STAT3 and autophagosome-lysosome fusion.

### Targeting UCHL3 protects against neuronal proteotoxic stress

**Figure 3:**
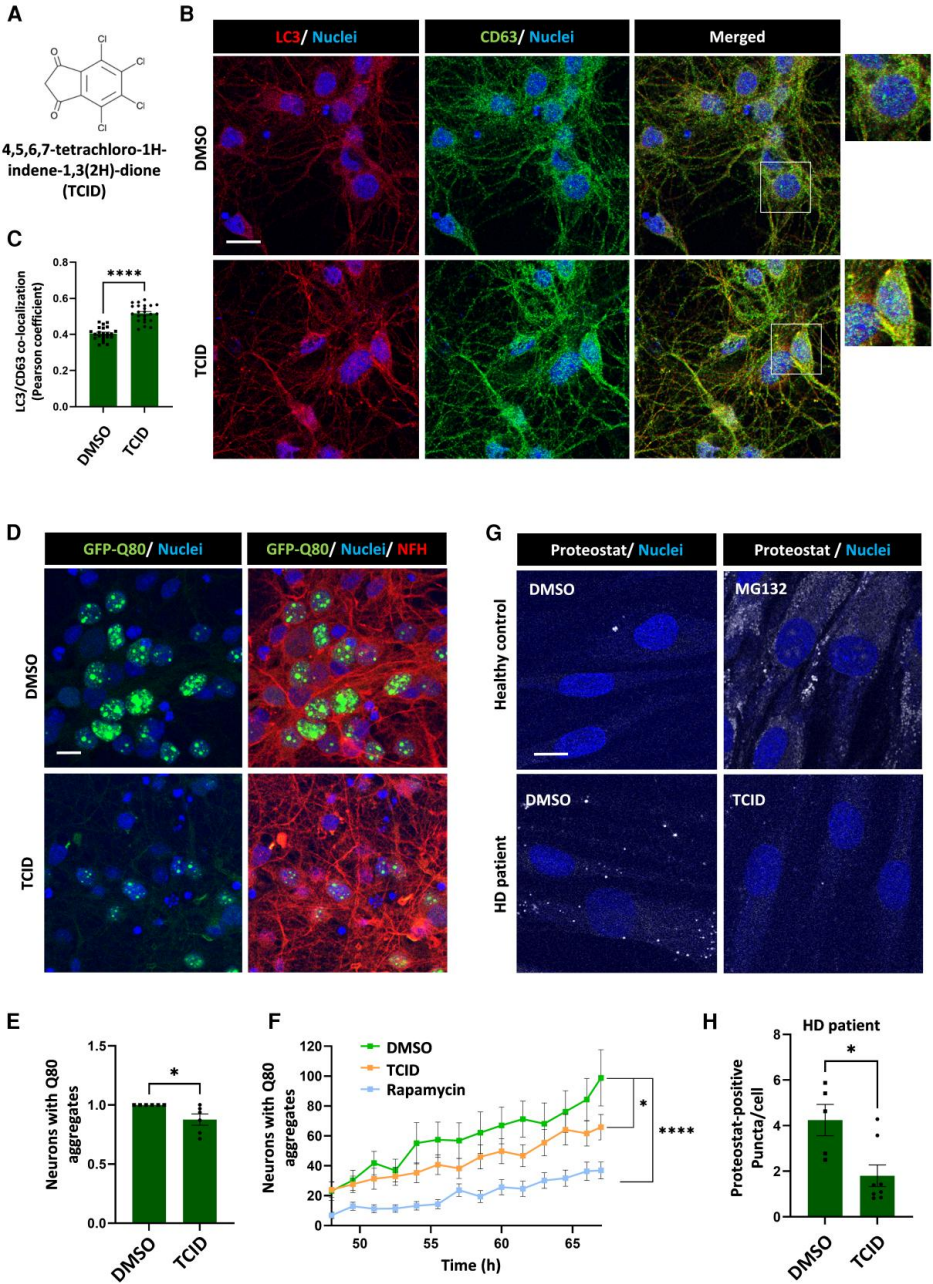
**TCID treatment of primary neurons and patient fibroblasts reduces proteotoxic stress caused by aggregate-prone proteins.** (**A**) The chemical structure of TCID. (**B** and **C**) Quantification of the co-localization of LC3 (red) and CD63 (green) by Pearson correlation in control DMSO and TCID (20 µM) treated mouse primary cortical neurons. Pearson correlation scores were taken from 3 independent cultures, *n* = 45 image fields were analysed each containing 5–10 neurons. (**D** and **E**) Quantification of nuclear polyQ aggregates in neurons transduced with lentiviruses encoding GFP-Q80 and treated with TCID. Data represent means ± SEM in *n* = 6 independent neuronal cultures. (**F**) Time-lapse imaging of GFP-Q80 aggregate formation in TCID and rapamycin (50 nM)-treated neurons. At least 10 000 cell bodies were sampled for the analysis. Results represent means ± SEM fraction of neurons with aggregates. *t* = 0 is defined as the viral transduction time. (**G** and **H**) Primary fibroblasts derived from a healthy control and Huntington’s disease (HD) patient were treated with either proteasome inhibitor (MG132) or TCID. The cells were stained with the proteostat dye and proteostat-positive puncta per cell was quantified in different image fields in the DMSO and TCID-treated HD fibroblasts. *n* = 13 fields each containing 15–25 cells were quantified. All images, scale bar = 20 μm. (**A**, **G** and **H**) Student’s *t*-test. (**F**) Two-way ANOVA and *post hoc* Sidak’s test. *P < 0.05, ****P < 0.0001. SEM = standard error of the mean.

We next examined whether pharmacological inhibition of UCHL3, which is used in cancer research, could be relevant in neurons. For this purpose, we used the UCHL3 small molecule inhibitor, TCID (Fig. 3A). Exposure of the primary neurons to TCID over a period of 72 h resulted in an increase in autophagosome-lysosome fusion detected by the LC3/CD63 double-labelled vesicles (Fig. 3B and C). TCID treatment significantly reduced the formation of intranuclear GFP-Q80 aggregates in neurons (Fig. 3D–F). The autophagy-inducing agent, rapamycin was used as a positive control in these experiments. Furthermore, we assessed protein aggregation in the healthy control and HD patient-derived primary fibroblasts. Fibroblasts were matched by sex and age, and the Proteostat dye was used to recognize aggregates from a wide spectrum of protein substrates. The results from normal control fibroblasts indicated that aggregation increases when the proteasome is inhibited (Fig. 3G). Interestingly, the HD cells exhibited more aggregate staining than control cells (even with no proteasome inhibition) that was significantly reduced after TCID treatment (Fig. 3G and H). TCID significantly reduced the levels of endogenous polyQ-expanded HTT in the HD fibroblasts by using an antibody that preferentially detects the mutant form of the protein (Supplementary Fig. 2A). Both UCHL3 depletion and TCID exposure in HD patient fibroblasts elevated STAT3 levels, and its nuclear localization is compatible with our model (Supplementary Fig. 2B and C). Finally, we treated the HD fibroblasts with Stattic, a small-molecule inhibitor of STAT3 activation. Stattic treatment to the cells prevented TCID-mediated localization of LC3/CD63 vesicles (Supplementary Fig. 2D), suggesting that STAT3 activation regulates TCID effects on autophagy.

### The protective effects of UCHL3 extend to human neurons vulnerable in HD

To examine whether UCHL3 could be a relevant target in brain regions affected in HD, UCHL3 expression was analysed in association with HTT in the brain striatum. The striatal brain sections derived from zQ175 HD mice (homozygous knock-in HTT mutation of 175Q) at 7 months of age showed UCHL3 association with striatal cells positive to HTT aggregates (Supplementary Fig. 1D and E). Indeed, proximity ligation assay targeting endogenous protein-protein interactions demonstrated that a distinct pool of UCHL3 is spatially associated with HTT; the autophagy-initiating kinase ULK1 was employed as a negative control to confirm the specificity of the interaction (Supplementary Fig. 3).

**Figure 4:**
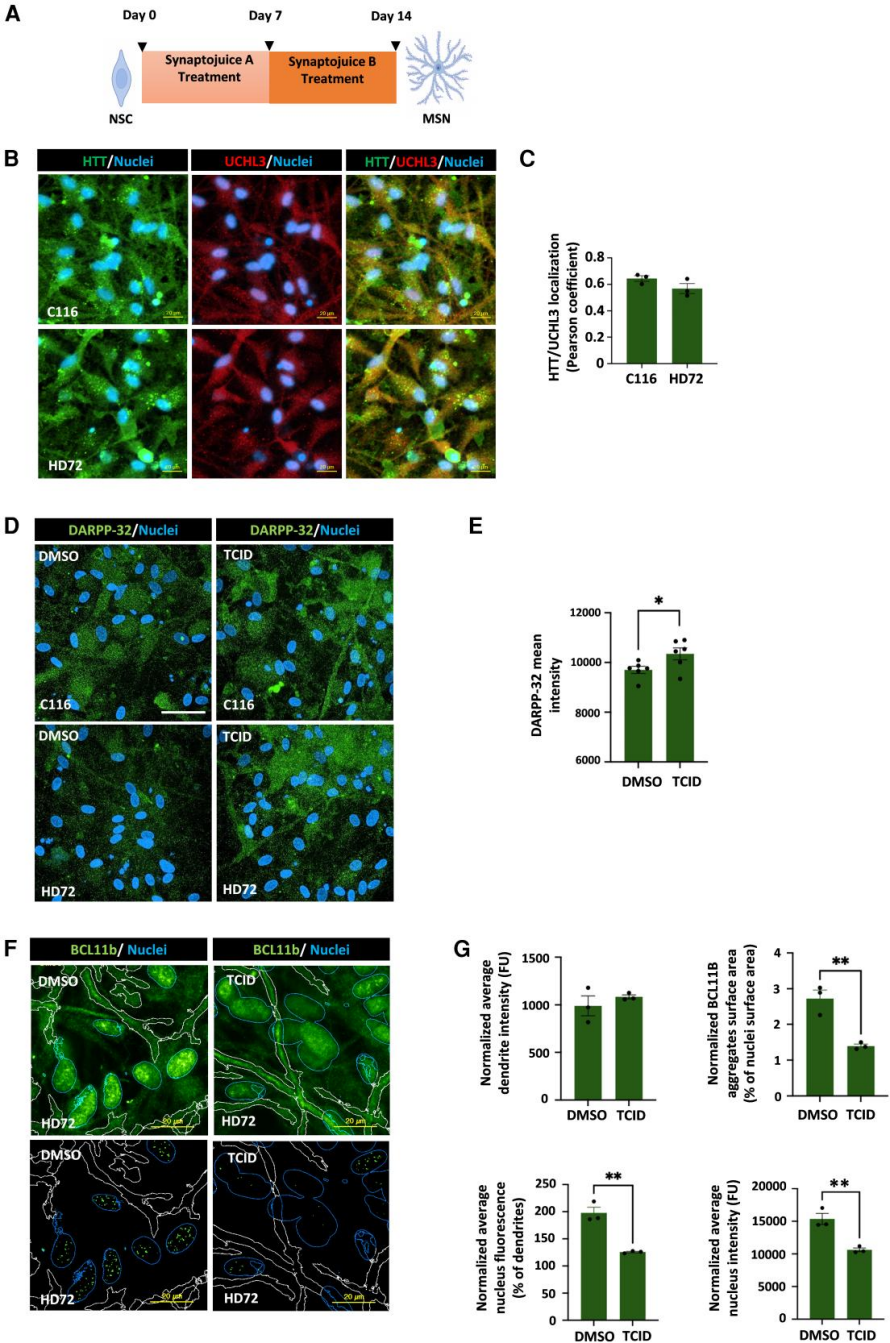
**UCHL3 inhibition protects against Huntington’s disease-related toxicity in medium spiny neurons.** (**A**) Schematic of human neural stem cell (NSC) differentiation into medium spiny neurons (MSN). (**B** and **C**) HTT (green) and UCHL3 (red) immunofluorescence staining of C116 and HD72 MSN (scale bar = 20 μm). Pearson’s correlation coefficient was performed to measure the co-localization of HTT and UCHL3 in MSN (*n* = 3 experiments). (**D** and **E**) The immunofluorescence image of DARPP-32 (green) stained C116 and HD72 MSN with and without TCID treatment (20 µM). Image scale bar = 50 μm. Quantification shows the mean intensity after TCID treatment. (**F** and **G**) Immunofluorescent staining for BCL11b (green) in HD72 MSN treated with TCID. Image scale bar = 20 μm. Quantification shows BCL11b aggregate surface area percentage, average nucleus intensity, average dendrite intensity, and average nuclear fluorescence as a percentage of dendrites (*n* = 3 experiments). (**E** and **G**) Student’s *t*-test. *P < 0.05, **P < 0.01. C116 = isogenic control (21Q/19Q); HD72 = HD patient-derived iPSCs (72Q/19Q).

The selective loss of striatal MSN is a key phenotype in HD but it is imperfectly recapitulated in mouse models.^4,25^ Therefore, to directly assess the role of UCHL3 in the loss of MSN, we used HD patient-derived iPSCs (72Q/19Q, termed HD72) and isogenic control (21Q/19Q, termed C116). Both iPSCs were differentiated into MSN by our established protocol of neuronal induction, regional patterning towards a lateral ganglionic eminence identity, and terminal differentiation (Fig. 4A). Compatible with the mouse data, the C116 and HD72 MSN showed co-localization between UCHL3 and HTT (Fig. 4B and C). We exposed the MSN to TCID and analysed (i) BCL11B as a pan-MSN marker; and (ii) DARPP-32 that is expressed later in development.^25^ Since low levels of DARPP-32 is a known signature of HD pathology, we thought to examine its modulation by UCHL3 inhibition. Notably, DARPP-32 levels were increased in the HD72 MSN following TCID treatment (Fig. 4D and E). BCL11B is essential for the development of corticospinal motor neuron projections and the differentiation of MSN.^25,26^ We have recently described that mislocalization of BCL11B into nuclear aggregates in HD MSN reflects a mechanism for BCL11B loss of function contributing to MSN reduced viability.^27^ The analysis of aggregates in nuclei and in dendrites revealed that treatment of TCID lowered the levels of BCL11B aggregates (Fig. 4F and G). These findings indicate that UCHL3 suppression attenuates disease-relevant markers in multiple cell types affected in HD.

## Discussion

One of the key areas of research focus in the search for disease-modifying therapies for HD is the alleviation of the proteotoxic stress caused by aggregate-prone polyQ expanded proteins.^11^ The proteostasis module of protein degradation via autophagy and the ubiquitin-proteasome system (UPS) plays a crucial role in the clearance of aggregate-prone proteins and in promoting neuronal survival under conditions of proteotoxic stress.^1,11^ DUBs can function in both the UPS and in regulating autophagy,^12^ highlighting this class of ubiquitin enzymes as promising targets for the development of small molecule inhibitors.^28^ Among DUBs, UCHL3 functions are exclusively studied outside the CNS because of its association with certain cancers.^14,16,17,19^ This study elucidates UCHL3 functions in post-mitotic neurons of the CNS.

One of the underlying mechanisms described in this study is the upregulation of neuronal autophagy via increasing autophagosome-lysosome fusion. However, this measure alone does not fully establish an increase in autophagic flux, and further work will be required to quantify flux dynamics directly. Additional cell survival mechanisms are likely to be involved considering the diverse pathways impacted by UCHL3. Only a few molecular targets of UCHL3 have been described thus far. A key example, which is also relevant to cancer, is the deubiquitination of Aurora B by UCHL3 to control its levels for proper microtubule attachment during the prometaphase stage of mitosis.^24^ We provide evidence that STAT3, implicated in protection against neuronal proteotoxic stress, is regulated by UCHL3. The induction of STAT3 levels after the suppression of UCHL3 primarily occurs at the transcriptional level and is likely affected by UCHL3 regulatory modules in the nucleus. STAT3 was previously described as a modulator of autophagy via the transcriptional regulation of several autophagy-related genes.^8^ In the context of HD, *STAT3* mRNA reduction, due to miR-29b-3p, promotes degeneration of HD patient-derived MSN.^8^ Therefore, suppression of UCHL3 may counteract the decline in *STAT3* mRNA and improve STAT3 function in HD-affected neurons, potentially providing a mechanistically distinct route from canonical mTOR-directed strategies for autophagy enhancement.

Although expansions beyond 36 repeats are typically considered pathological in HD, other factors, such as progression of disease, may be impacted by the length of the repeat. Additional neuropathologic study of post-mortem human HD brains and controls is warranted to reveal the relationship between UCHL3, STAT3 and autophagy markers across a range of CAG repeat expansions and thus different HTT inclusion densities. Moreover, while TCID served as a useful pharmacological probe, the current evidence does not yet define its selectivity among other DUB families or confirm direct target engagement in neurons. Furthermore, its pharmacokinetic and safety profiles, including brain exposure, remain to be established.

Cancer and neurodegeneration are often considered opposite diseases in terms of their underlying mechanisms. Cancer is characterized by uncontrolled cellular proliferation, whereas neurodegenerative diseases are marked by progressive cellular atrophy and death. However, many signalling pathways are commonly affected in both diseases. For example, there is a reduced cancer rate in patients with polyQ diseases (excluding skin cancer) despite the high incidence of risk factors.^29^ Nilotinib, an FDA-approved drug for the treatment of chronic myelogenous leukaemia, was recently examined in a phase 1 clinical trial in HD patients.^30^ Overall, this study highlights UCHL3 as a previously unrecognized modulator of neuronal proteostasis, suggesting that mechanistic lessons from cancer biology may inform the discovery of potential targets for modulating HD and related proteinopathies.

## Supplementary Material

awag028_Supplementary_Data

## Data Availability

The proteomic DAVID dataset used in this study is listed in the Supplementary material. Further requests for resources and information should be directed to the corresponding author.
