# Supplementary material for: Targeting UCHL3 attenuates pathological markers in neuronal models of Huntington’s disease
Source: Brain. 2026 Jan 24;149(6):1893–901. doi: 10.1093/brain/awag028 (PMC13232036; doi:10.1093/brain/awag028)

Supplementary material (Ishtayeh et al.)

Supplementary methods

Ethics statement

All mouse experiments were reviewed and approved by the Institutional Animal Care and Use Committees of Tel Aviv University and the Buck Institute. All cell lines, primary human cells and protocols related to human stem cell research in the present study were used in accordance with guidelines approved by the Institutional Review Board.

HD patient-derived MSN differentiation

C116 and HD72 neural stem cells (NSC) were generated as follow: plates were treated with Matrigel ON at 37°C in 96-well plates (40,000-60,000 cells/well). Two differentiation media were prepared. The first media, Synaptojuice A (SJA) contained 10X synaptojuice A supplement (5 mL), advanced DMEM/F12 medium (44.1 mL), penicillin/streptomycin (P/S) (450 µL), and 100X Glutamax (450 µL). Synaptojuice A supplement (10X) contains advanced DMEM/F12 medium (38 mL), MACS NeuroBrew-21 with retinoic acid with final concentrations noted (10 mL), DAPT (100 µM), PD0332991 (20 µM), human brain-derived neurotrophic factor (BDNF, 100 ng/mL), LM22A4 (5 µM), forskolin (100 µM), gamma-aminobutyric acid (GABA, 3 mM), CHIR 99021 (30 µM), $CaCl_2$ (1.8 mM), ascorbic acid (2 mM). The second differentiation media, Synaptojuice B (SJB), was prepared with 10X synaptojuice B (5 mL) supplement and basal medium (45 mL). Basal medium contains advanced DMEM/F12 medium (22.5 mL), and 100× Glutamax (450 µL), P/S (450 µL), and Neurobasal A Medium (22.5 mL). Synaptojuice B supplement contains advanced DMEM/F12 medium (19.7 mL), Neurobasal A medium (19.7 mL), PD0332991 (100 µL, 20 µM), human BDNF (50 µL, 100 ng/mL), MACS NeuroBrew-21 with retinoic (10 mL), LM22A4 (25 µL, 5 µM), CHIR 99021 (250 µL, 30 µM), GABA (500 µL, 3 mM), $CaCl_2$ (370 µL, 1.8 mM), and ascorbic acid (100 µL, 2 mM).

Production and infection of lentivirus

The RNAi Consortium (TRC) lentiviral pLKO.1-puro-mouse UCHL3 shRNA vectors were purchased from MERCK. The sequences of the U6 promoter driven anti-UCHL3 shRNAs were:

5'- CCGGCCTGAACTTCTTAGCATGGTACTCGAGTACCATGCTAAGAAGTTCAGGTTTTTG –3'

5'–CCGGCCCTGATGAGTTAAGATTTAACTCGAGTTAAATCTTAACTCATCAGGGTTTTTG – 3'

for TRCN0000030715 and TRCN0000030717 respectively. Lentiviral vector expressing Q80-EGFP fusion protein under the control of the neuron-specific synapsin promoter was previously

described [1, 2]. We have previously described the generation of lentivirus particles with helper plasmids pMDLg/pRRE, pRSV-Rev, and pCMV-VSVG that carry HIV regulatory protein genes, as well as the pseudotyped envelope protein gene from vesicular stomatitis virus envelope G (VSV G) [3].

Mouse primary neurons

Cortical neurons were cultured from wild-type C57BL/6J mouse embryos at E17 as we have previously described [3]. After extraction, brains were placed in cold Hanks' Balanced Salt Solution (HBSS) and the cortices were isolated under a dissection microscope. The cortices were then dissociated and seeded on plates coated with poly-d-lysine in neurobasal media supplemented with 1% GlutaMAX™ Supplement, 1% Sodium pyruvate, 2% B27 supplement and 1% Penicillin–Streptomycin. Prior to treatment, half of the culture media was changed every 3 days. Lentiviral transduction was followed by media change after 24 hours and then incubation for 72-96 hours.

HD patient-derived primary fibroblasts and cell lines

Primary skin fibroblasts from a female age 37 with severe HD symptoms (*HTT*: 50 CAG repeats/15 CAG repeats) and from age-matched healthy control female were obtained from The Coriell Institute for Medical Research. The primary cells were cultured in full DMEM media consisting of 10% heat-inactivated FBS, 1% PS, 1% L-Glutamine, 1% NEAA and 1% pyruvate. HeLa cell line was purchased from ATCC (line CCL-2). The cells were cultured in full DMEM media, consisting of 10% FBS, 1% PS and 1% L-Glutamine. Cells were cultured at 37°C in 5% CO_2 and were routinely tested for mycoplasma.

Immunohistochemistry of WT and homozygous zQ175 brain

WT and zQ175 homozygous mice brains were collected at 7 months of age and perfused with paraformaldehyde. Deparaffination was performed with two incubations of xylene (7 m), 2 with 100% ethanol (2 m), 2 with 95% ethanol (2 m), 1 with 80% ethanol (4 m) and 1 with 70% ethanol (4 m). Antigen retrieval was performed with citrate buffer at pH 6 for 30 m. Slides were blocked with 10% bovine serum albumin, 1% normal donkey serum, 0.1% Triton X-100. Slides were incubated with primary antibodies mouse anti-HTT antibody, clone mEM48 (1:100) and rabbit anti-UCHL3 antibody (1:100) at 4°C ON. After washing with PBS, slides were incubated with secondaries (647 donkey anti-rabbit, 488 donkey anti-mouse, 1:2000) for 1 h. After washing with PBS, slides were incubated with DAPI and True Black Lipofuscin Autofluorescence Quencher (Biotium) before mounting with Prolong Gold Antifade Mountant and glass coverslips. Pearson's

correlation in histological sections was calculated in Image Analyst MKII by manually drawing ROIs on select areas of the images and using the “Plot Correlation (Colocalization) function”.

Treatment with small molecules

The treatment protocol in cortical neurons: aliquots of TCID dissolved in DMSO for a concentration of 20 mM were diluted 1:1000 for a final concentration of 20μM in media and incubated for a period between 48-96 hours with the neurons, replacing the media with fresh media with TCID at the 24-hour mark. Treatment protocol in HD MSN: NSCs were seeded on a 96-well plate and left at 37°C ON. Neurobasal media was completely aspirated and replaced with 200 μL SJA + Activin A (AA). Half (100 μL) of SJA media was removed on alternating days and replaced with fresh SJA + AA. A total of 5 rounds of SJA treatments were completed. SJA was aspirated completely and replaced with SJB + AA. A total of 7 rounds of SJB treatments were completed. Treatment of TCID accompanied the third day of SJB treatment at three different concentrations (10, 20, 40 μM) to evaluate optimal drug concentration, with a total of 5 TCID treatment days.

Immunostaining and imaging analysis

Primary neurons, fibroblasts, and cell lines were cultured on coverslips for mounting on glass slides. The cells were washed with warm PBS and fixed in 4% formaldehyde PBS solution followed by incubation with 0.1% TritonX100 in PBS. The coverslips were then incubated with a 2% BSA in PBS Blocking solution for 1 h then incubated overnight with a primary antibody (1:150). Afterwards, coverslips were washed and incubated with secondary fluorescent antibodies (1:300- 1:1000). The cells were then washed again and incubated with DRAQ5 solution with PBS. The coverslips were then mounted on microscope slides with mounting medium, Fluoro-Gel II with DAPI. The slides were allowed to dry for up to 24 h, then stored at 4°C, then imaged on a Zeiss 710 confocal microscope. The Duolink® Proximity Ligation Assay kit for protein-protein interaction was used according to manufacturer's instructions. The PROTEOSTAT® Aggresome detection kit was used to detect aggregated and misfolded proteins. ImageJ was used to quantify the puncta following thresholding and using the analyze particles feature. To find the Pearson coefficient for LC3, CD63 co-localization, imaging fields (n=23-24) were analyzed from independent neuronal cultures. The ImageJ plugin JaCoP was used to analyze the images. For STAT3 nuclear intensity, the “analyze particles” function was used on the DAPI channel to isolate nuclear areas, then the STAT3 channel was measured in those areas to give the intensity of STAT3 in the nucleus. Results represent means of nuclear intensity of STAT3 in different image fields

from independent neuronal cultures. For the quantification of nuclear polyQ aggregates, results are from independent neuronal cultures, each with quantified cells n=250-350. Aggregates were counted manually during imaging.

For HD MSN cultures: Plates were fixed with 4% paraformaldehyde for 12 m (wash 3X with PBS). Plates were blocked for 1 h on shaker (100 µL Triton X-100 + 4 mL NDS + 96 mL PBS). Plates were incubated with primary at 1:100 concentration ON at 4°C (mouse anti-Darpp-32 (H-3) and rabbit anti-BCL11B). Plates were washed 3X with wash buffer (100 µL Triton X-100 + 100 mL PBS) and incubated with secondary (1:500) for 2h at RT. Plates were washed 3X with wash buffer and incubated with DAPI for 1 m. Plates were imaged on a Biotek Cytation 5 at 40X magnification and a Revvity Operetta CLS spinning disc confocal microscope using a 20X NA water immersion lens at 0.6µm/pixel resolution and z-stacking. 3X3 tiled view fields were recorded in quadruplicate for each condition.

For live-cell imaging, primary cortical neurons were seeded into 96-well plates. Seeding confluency was 60,000- 90,000 cells per well. The Q80-GFP lentivirus was administered three days after initial seeding (defined as time=0). Treatment with TCID (20µM) and Rapamycin (50nM) was administered two days after the lentiviral transduction. Following drug treatment, the plates were placed in the Incucyte® Live-Cell Analysis System to monitor Q80-GFP aggregate formation over time. Incubation and imaging lasted for 48-72 hours after which imaging data was extracted for analysis. Green fluorescent images were acquired to identify and measure Q80-GFP expression and aggregation. Quantification of cell number and aggregate number was done using the Incucyte integrated analysis software. For calculating data points, the rate of aggregation was calculated for each well. The number of cells containing GFP aggregates was divided by the number of total cells expressing the Q80-GFP. A baseline aggregation rate of the control condition was defined for each experiment which was then used to normalize aggregation rates across multiple experiments.

Analysis of BCL11B aggregates in nuclei and in dendrites of MSN

Two-channel confocal microscopic recordings of BCL11b immunofluorescence and DAPI were analyzed in Image Analyst MKII and FIJI, with the combination of morphological nuclei segmentation and Labkit pixel classification. The percent of nucleus area occupied in a single confocal plane by BCL11B aggregates was measured similarly as previously described for mitochondria:cell volume fraction [4]. Nucleus profiles were determined by global thresholding.

Dendrite profiles were determined by training a pixel classifier in Labkit [5]. Overlapping detection of nuclei and dendrites were excluded from analysis, and then BCL11B immunofluorescence intensity was measured over dendrites and nuclei, and the percent area over nuclei (see pipeline "Measure intranuclear aggregates and stain intensities in nuclei and dendrites with Labkit segmentation" at https://github.com/gerencserlab/IA-Protein-Aggregates.git).

mRNA analysis by RT-qPCR

The qScript cDNA Synthesis Kit and qPCRBIO SyGreen Blue Mix Hi-ROX kit RT-qPCR were used for real-time q-PCR analysis. Primers used for RT-qPCR for STAT3: FORWARD 5'-GCTCTCCCACCCCATCAAT-3', REVERSE 5'-AAATACCCTTTCAGTTTATAA-GTTCAGCTT-3'. Actin was used as a housekeeping gene. Primers for Actin used were as follows: FORWARD 5'-CCTGAACCCTAAGGCCAACC-3' REVERSE 5'- ATGGCGTGAGG-GAGAGCATA-3'.

Isolation of cytoplasmic and nuclear extracts

To separate nuclear and cytoplasmic fractions, neurons were collected from 10 cm plates by cell scraper into 500 µL of IP lysis buffer with Protease Inhibitor Cocktail cOmplete™ and incubated on ice for 15 min. Mechanical lysis was achieved by passing the suspension through a 27-gauge needle ten times to ensure complete disruption of cell membranes, followed by an additional 20-min incubation on ice. The lysate was centrifuged at 720 × g for 5 min, resulting in a nuclear pellet and a supernatant containing cytoplasmic proteins. The nuclear pellet was washed with 500 µL buffer, re-suspended using a pipette, and further homogenized by passing through a 25-gauge needle ten times. The supernatant for the nuclear pellet was discarded and the pellet was re-suspended and briefly sonicated, **3 to 10 seconds per pulse** at **low power settings.** The cytoplasmic fraction, present in the supernatant from the initial centrifugation, was collected and kept on ice.

Western blot

Cells were harvested in sample buffer x2 containing 5% 2-mercaptoethanol or with IP lysis buffer. Lysates were then boiled at 95 °C for 5 min and vortexed. Next, lysates were subjected to electrophoresis on 10-15% SDS-PAGE gels. Proteins were transferred to 0.2 µm PVDF membranes which underwent blocking with PBST (0.1% Tween 20) buffer with 5% non-fat milk for 1 hour then incubated overnight with primary antibodies. After the incubation, the membranes were washed and incubated with the appropriate secondary antibody for mouse or rabbit-derived antibodies. Antibodies were then washed again and developed using a WESTAR NOVA 2.0

chemiluminescent kit. Chemical luminescence of membranes was detected using the UVITEC ALLIANCE (Uvitec, England). The intensity of protein bands was then quantified by ImageJ.

Proteomic analysis

HeLa cells were transfected with a vector containing the FLAG- and HA tags fused to the human UCHL3 (Addgene Plasmid #22564 [6]) or a control empty vector. After 48h post transfection, the cells were harvested with immunoprecipitation (IP) lysis buffer (20 mM Tris-HCl, pH 7.2, 150 mM NaCl, 2 mM $MgCl_2$, 0.5% NP-40) and immunocomplexes were pulled down with mouse anti-HA antibody coupled to agarose beads. The beads were boiled for 5 min at 95°C, and proteins were resolved and digested in-gel. The resulting tryptic peptides were analyzed by Q Exactive LC-MSMS. Raw files were processed using the DiANN identification and quantification against human proteome from the UniProt database. Functional pathway clustering of protein hits (with coverage of two or more unique peptides) enriched in the UCHL3 pulldown against control were analyzed using DAVID (v2022q3 release) [7].

Supplementary Tables

Supplementary Table 1. Reagents and experimental models used in this study and their source/ catalogue numbers.

Reagent/Resource	Reference or Source	Identifier or Catalogue Number
**Experimental Models**		
C57BL/6J (*M. musculus*)	Amer-Sarsour et al.[3]	
zQ175 (*M. musculus*)	JAX	Strain #370476
Fibroblasts (HD)	Coriell Institute	GM04687
Fibroblasts (Healthy)	Coriell Institute	GM01650
HD neural stem cells	Tshilenge et al. [8]	HD72
Isogenic corrected neural stem cells	Tshilenge et al. [8]	C116
mRNA of HD brains	Labadorf et al. [9]	GSE64810
**Antibodies**		
Rabbit anti-GFP antibody	Abcam	ab6556
Rabbit anti-actin antibody	MERCK	A2066
Rabbit anti-UCHL3 antibody	Abcam	ab126621
Rabbit anti-STAT3 antibody	Abcam	ab68153
Rabbit anti-Lamin antibody	Abcam	ab16048
Mouse anti-GAPDH antibody	Abcam	ab8245
Rabbit anti-ULK1 antibody	Cell Signaling	8054S
Rabbit anti-BCL11B antibody	Novus Biological Littleton	NB100-79809
Mouse anti-Darpp-32 (H-3) antibody	Santa Cruz Biotechnology	SC-271111
Mouse anti-Huntingtin antibody (clone mEM48)	Millipore	MAB5374
Mouse anti-Huntingtin antibody	Millipore	MAB5490
Mouse anti-Polyglutamines antibody	MERCK	P1874

Rabbit anti-NFH antibody	MERCK	N4142-.2ML
Rabbit anti-LC3 antibody	Abcam	ab192890
Rabbit anti-CD63 antibody	Santa Cruz Biotechnology	sc15363
Mouse anti-HA antibody	Biolegend	901502
Goat Anti-Mouse IgG H&L (HRP)	Abcam	ab205719
Goat Anti-Rabbit IgG H&L (HRP)	Abcam	ab6721
Goat Anti-Mouse IgG H&L (Alexa Fluor® 555)	Abcam	150114
Goat Anti-Rabbit IgG H&L (Alexa Fluor® 555)	Abcam	150078
Goat Anti-Mouse IgG H&L (Alexa Fluor® 488)	Abcam	150113
Goat Anti-Rabbit IgG H&L (Alexa Fluor® 488)	Abcam	ab150077
Donkey Anti-Rabbit IgG HRP linked whole antibody	Millipore Sigma	NA934V
Anti-mouse IgG HRP linked whole antibody	Millipore Sigma	NXA931V
**Oligonucleotides and other sequence-based reagents**		
Mus.STAT3	Fatih et al. [5]	F: 5′-GCTCTCCCACCCCATCAAT-3’
Mus.STAT3	Fatih et al. [5]	R: 5′-AAATACCCTTTCAGTTTATAAGTTCAGCTT-3’
Mus.ACTIN	This study	F: 5′-CCTGAACCCTAAGGCCAACC-3’
Mus.ACTIN	This study	R: 5′-ATGGCGTGAGGGAGAGCATA-3’

**Chemicals, Enzymes and other reagents**		
HBSS	Thermo Fisher Scientific	1402550
poly-d-lysine	MERCK	P6407-5MG
Neurobasal Media	Thermo Fisher Scientific/ Gibco	12349015/ 10888022
GlutaMAX™	Thermofisher/ Invitrogen	35050-061/35050079
Sodium pyruvate	Thermo Fisher Scientific	11360039
B27	Thermo Fisher Scientific	17504044
Penicillin–Streptomycin	Sartorius/invitrogen	03-031-1B/15140122
Penicillin-Streptomycin	MERCK	P4333-100ML
Fetal Bovine Serum	Sartorius	04-007-1A
L-Glutamine	MERCK	G7513-100ML
TCID	Tocris Bioscience	5179
NEAA	Thermo Fisher Scientific	11140-035
Sodium Pyruvate	Thermo Fisher Scientific	11360-039
PROTEOSTAT® Aggresome detection kit	Enzo Life Sciences	ENZ51035
DRAQ 5 solution	Abcam	108410
DAPI-containing mounting medium	Electron Microscopy Sciences	17985-50
cOmplete™	MERCK	4693132001
qScript cDNA Synthesis Kit	Quanta BioSciences	95047-100-2
qPCRBIO SyGreen Blue Mix Hi-ROX kit RT-qPCR kit	PCRBiosystems	PB20.16-05
Activin A	PeproTech	AF-120-14E
Matrigel	Corning	CB-40234
Advanced DMEM/F12 Medium	Thermo Fisher Scientific	12634010
NeuroBrew-21 with retinoic acid	MACS Miltenyi Biotec	130-093-566
DAPT	Tocris Bioscience	2634
PD0332991	Tocris Bioscience	4786
Human BDNF	MACS Miltenyi Biotec	130-096-286
LM22A4	Tocris Bioscience	4607
Forskolin	Tocris Bioscience	1099
GABA	Tocris Bioscience	344
CHIR 99021	Tocris Bioscience	1099

$CaCl_2$	Tocris Bioscience	3148
Ascorbic Acid	Tocris Bioscience	4055
MES SDS Running Buffer	Invitrogen	NP0002
NuPage 4-12% Bis-Tris Gel	Invitrogen	NP0341
NuPage Antioxidant	Invitrogen	NP0005
Luminol Enhancer Reagent	Thermo Scientific	1859698
Donkey Serum IHC stain	Jackson ImmunoResearch Laboratorie	017-000-121
Peroxide Solution	Thermo Scientific	1859701
DAPI	Thermoscientific	62248
Stattic	Abcam	ab120952
True Black Lipofuscin Autofluorescence Quencher	Biotium	23007
Prolong Gold Antifade Mountant	Thermoscientific	P36930
Duolink® Proximity Ligation Assay	MERCK	DUO92008, DUO92002, DUO92004
**Software**		
ImageJ/Fiji	https://imagej.net/software/fiji/	
Image Analyst MKII	https://www.imageanalyst.net/	
Graph Pad Prism	https://www.graphpad.com/	
CellProfiler	https://cellprofiler.org/	
Incucyte live-imaging software	https://www.sartorius.com/en/products/live-cell-imaging-analysis/live-cell-analysis-software	

Supplementary Table 2. Functional pathway enrichments of UCHL3 interactors performed by DAVID.

Table S2 is supplied as xls. file.

Supplementary movies

Supplementary Movie 1. Time-lapse imaging of GFP-Q80 aggregate formation in control neurons.

Supplementary Movie 2. Time-lapse imaging of GFP-Q80 aggregate formation in UCHL3 KD neurons.

Supplementary references

1. Jimenez-Sanchez, M. *et al.* siRNA screen identifies QPCT as a druggable target for Huntington's disease. *Nat Chem Biol* **11**, 347-354 (2015).
2. Howarth, J.L. *et al.* Hsp40 Molecules That Target to the Ubiquitin-proteasome System Decrease Inclusion Formation in Models of Polyglutamine Disease. *Mol Ther* **15**, 1100-1105 (2007).
3. Amer-Sarsour, F. *et al.* Disease-associated polyalanine expansion mutations impair UBA6-dependent ubiquitination. *EMBO J* **43**, 250-276 (2024).
4. Lerner, C.A. & Gerencser, A.A. Unbiased Millivolts Assay of Mitochondrial Membrane Potential in Intact Cells. *Methods Mol Biol* **2497**, 11-61 (2022).
5. Wang, G. *et al.* PyMIC: A deep learning toolkit for annotation-efficient medical image segmentation. *Comput Methods Programs Biomed* **231**, 107398 (2023).
6. Sowa, M.E., Bennett, E.J., Gygi, S.P. & Harper, J.W. Defining the human deubiquitinating enzyme interaction landscape. *Cell* **138**, 389-403 (2009).
7. Sherman, B.T. *et al.* DAVID: a web server for functional enrichment analysis and functional annotation of gene lists (2021 update). *Nucleic Acids Res* **50**, W216-221 (2022).
8. Tshilenge, K.T. *et al.* Proteomic Analysis of Huntington's Disease Medium Spiny Neurons Identifies Alterations in Lipid Droplets. *Mol Cell Proteomics* **22**, 100534 (2023).
9. Labadorf, A. *et al.* RNA Sequence Analysis of Human Huntington Disease Brain Reveals an Extensive Increase in Inflammatory and Developmental Gene Expression. *PLoS One* **10**, e0143563 (2015).

Supplementary figures

Supplementary Figure 1, Supplementary Figure 2, Supplementary Figure 3, Uncropped blots.

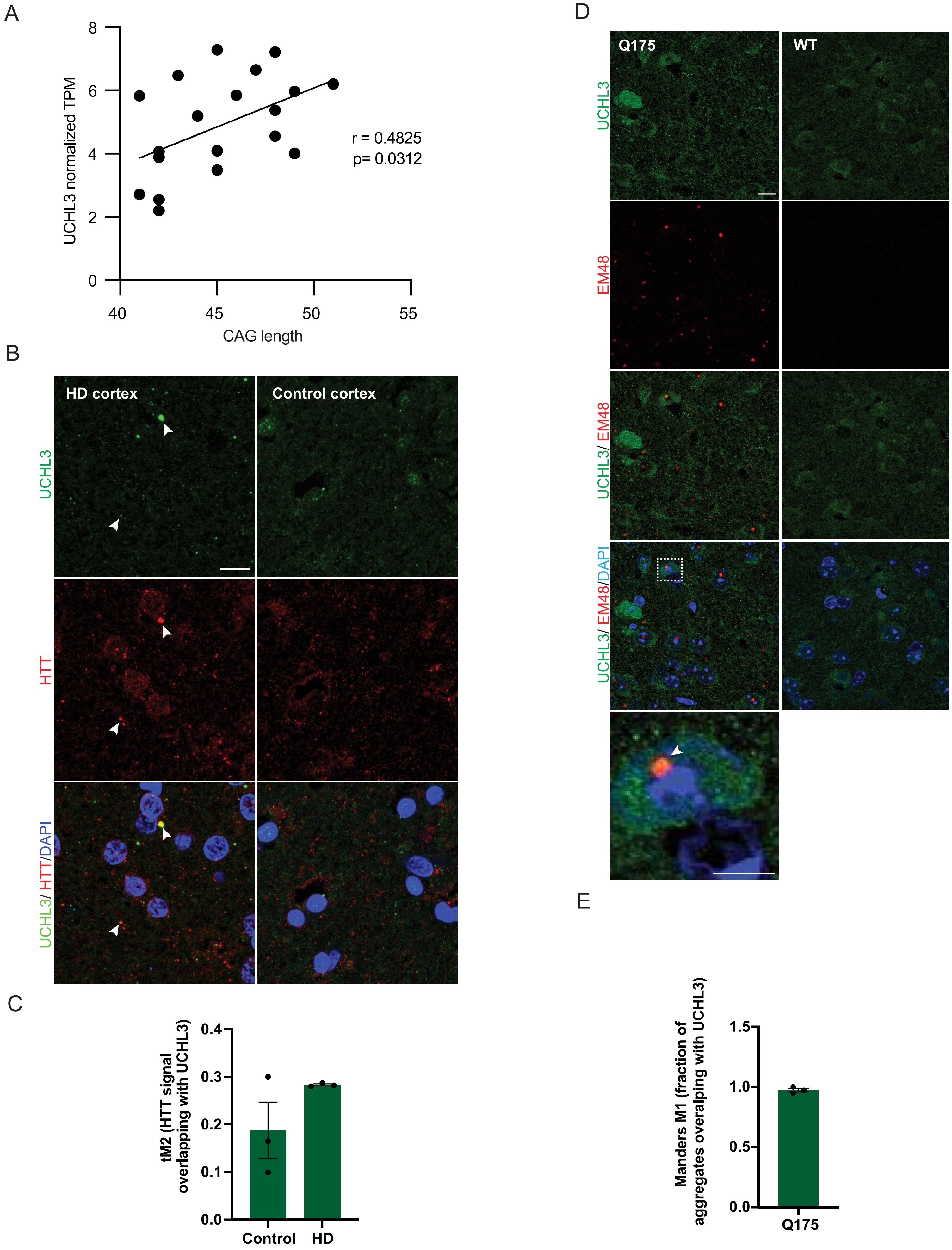
A
UCHL3 normalized TPM
CAG length
r = 0.4825
p= 0.0312
B
HD cortex
Control cortex
UCHL3
HTT
UCHL3/ HTT/DAPI
C
tM2 (HTT signal overlapping with UCHL3)
Control
HD
D
Q175
WT
UCHL3
EM48
UCHL3/ EM48
UCHL3/ EM48/DAPI
E
Manders M1 (fraction of aggregates overalping with UCHL3)
Q175

Supplementary Figure 1. UCHL3 expression correlates with CAG repeat length and colocalizes with HTT in the human HD cortex and mouse Q175 striatum. (**A**) Correlation between HTT CAG repeat length and normalized *UCHL3* transcript levels in postmortem human BA9 cortex from Huntington's disease patients. Expression values are from RNA-seq data in dataset GSE64810 (Labadorf et al., 2015, *PLoS One*). *UCHL3* normalized TPM values were plotted against CAG repeat length for HD individuals. Pearson correlation analysis revealed a significant positive association ($r = 0.4825$, $p = 0.0312$). (**B**) Representative confocal images showing UCHL3 and HTT (MAB5490) immunoreactivity in control and HD human cortex. Scale bar 10µm. (**C**) Quantification of the spatial overlap between HTT and UCHL3 signals in HD and control cortex. tM2 values reflect Manders' colocalization coefficients of HTT signal overlapping with UCHL3. (D) Representative confocal images from striatal sections of Q175 and WT mice stained for UCHL3 and EM48 (HTT aggregates). Q175 mice displayed abundant $EM48^+$ aggregates that colocalized with UCHL3, whereas WT showed no detectable $EM48^+$ aggregates. The inset highlights a representative $EM48^+$ aggregate colocalizing with UCHL3. (E) Quantification of Manders' M1 values showed that nearly all $EM48^+$ aggregates in Q175 mice colocalized with UCHL3. The Costes randomization test confirmed that the colocalization was not due to chance ($P > 0.95$). WT samples lacked EM48+ aggregates, and therefore Manders' M1 values could not be calculated. Scale bars, 10 µm (overview) and 5 µm (inset). Data are shown as mean ± SEM.

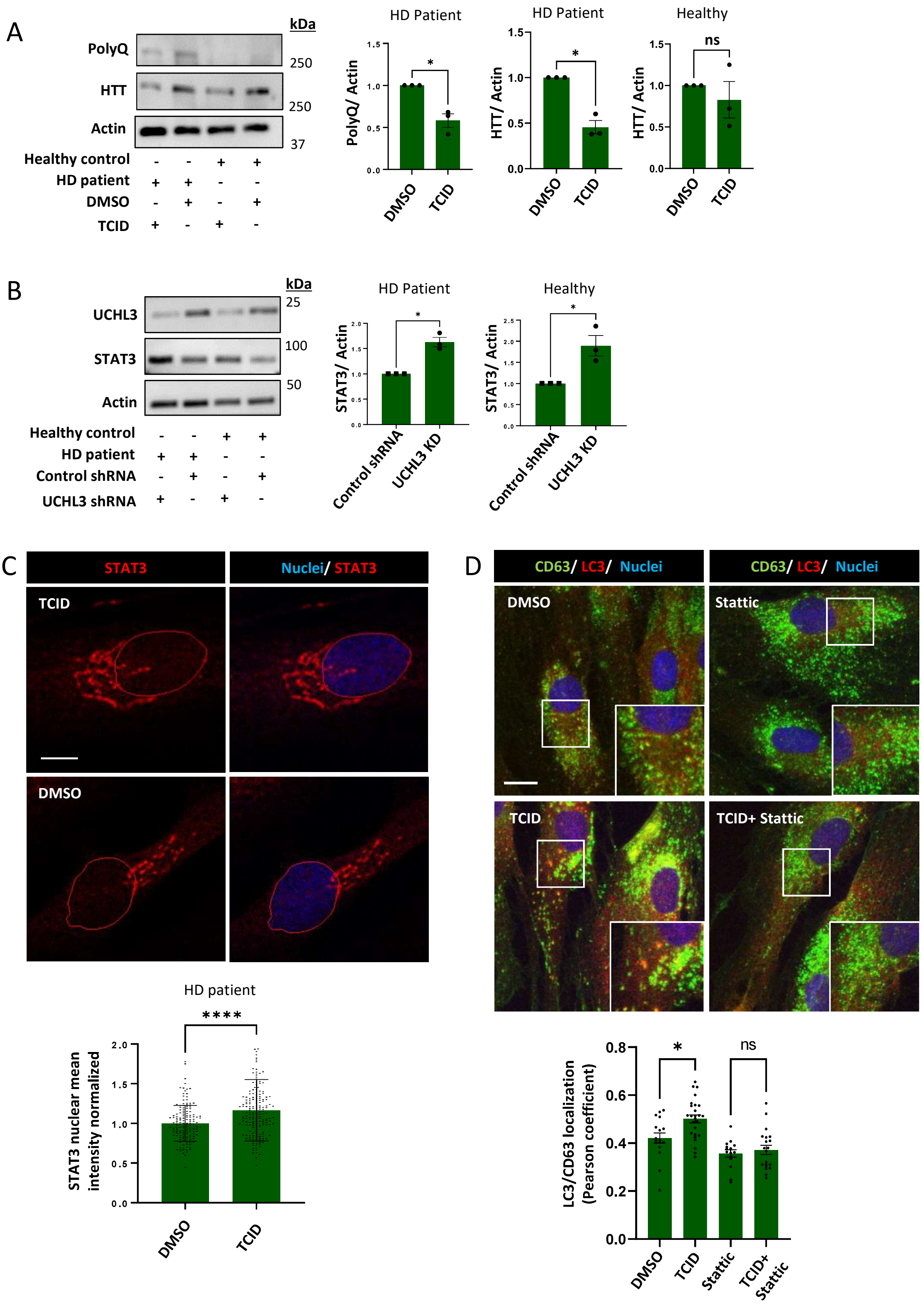

A
kDa
PolyQ
HTT
Actin
250
250
37
Healthy control
HD patient
DMSO
TCID
HD Patient
PolyQ/ Actin
HTT/ Actin
Healthy
ns
DMSO
TCID
B
UCHL3
STAT3
25
100
50
Control shRNA
UCHL3 shRNA
STAT3/ Actin
UCHL3 KD
C
STAT3
Nuclei/ STAT3
HD patient
****
STAT3 nuclear mean intensity normalized
D
CD63/ LC3/ Nuclei
Stattic
TCID+ Stattic
LC3/CD63 localization (Pearson coefficient)

Supplementary Figure 2. STAT3 and HTT regulation by UCHL3 suppression in HD patient fibroblasts. **(A)** HTT protein levels were quantified with anti-HTT and anti-PolyQ antibodies in control and HD patient-derived fibroblasts treated with TCID (20 µM) for 72 hours. **(B)** STAT3 and UCHL3 protein levels were quantified in scrambled control shRNA and UCHL3 KD primary HD patient-derived fibroblasts. (A-B) n=3 experiments. Data represents means ± SEM. **(C)** HD patient fibroblasts were treated with TCID for 48 hours and stained for STAT3. Image Scale bar 10 µm. Quantification of STAT3 intensity in individual nuclei (n=150). Data represents means ± SD. (**D**) Quantification of the co-localization of LC3 (red) and CD63 (green) by Pearson correlation in control DMSO and TCID-treated HD fibroblasts. At the last 4hr of the experiment, the STAT3 inhibitor, Stattic was added (2 µM). Scale bar 10 µm. Cells containing CD63 positive vesicles were analyzed for LC3 and CD63 co-localization in different image fields; n= 80 cells. A-C, Student's t test. D, one-way ANOVA and Tukey's post-hoc test. *p<0.05, ****p<0.0001.

Proximity ligation assay

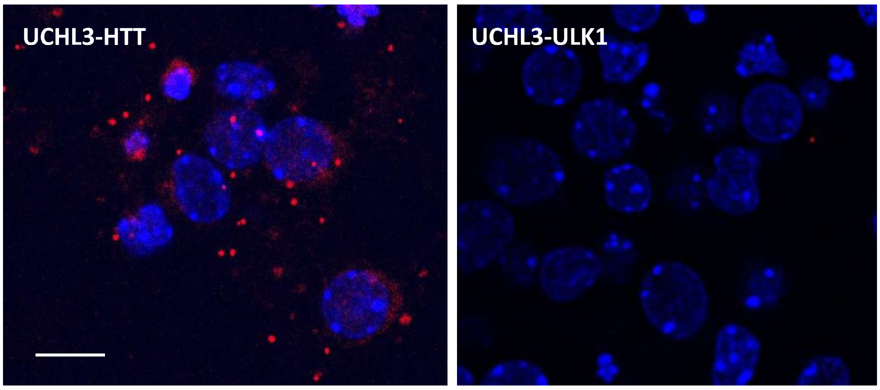

Supplementary Figure 3. Analysis of UCHL3 association with HTT. Mouse primary cortical neurons were fixed and analyzed by proximity ligation assay (PLA) using primary antibodies against HTT and UCHL3 or using primary antibodies against a control protein, ULK1 and UCHL3. Cells were imaged by confocal microscopy (scale bar 10 µm). The PLA yields a signal (labeled by red puncta) when two proteins are within 40 nm of each other.

Uncropped Blots

Figure 1D

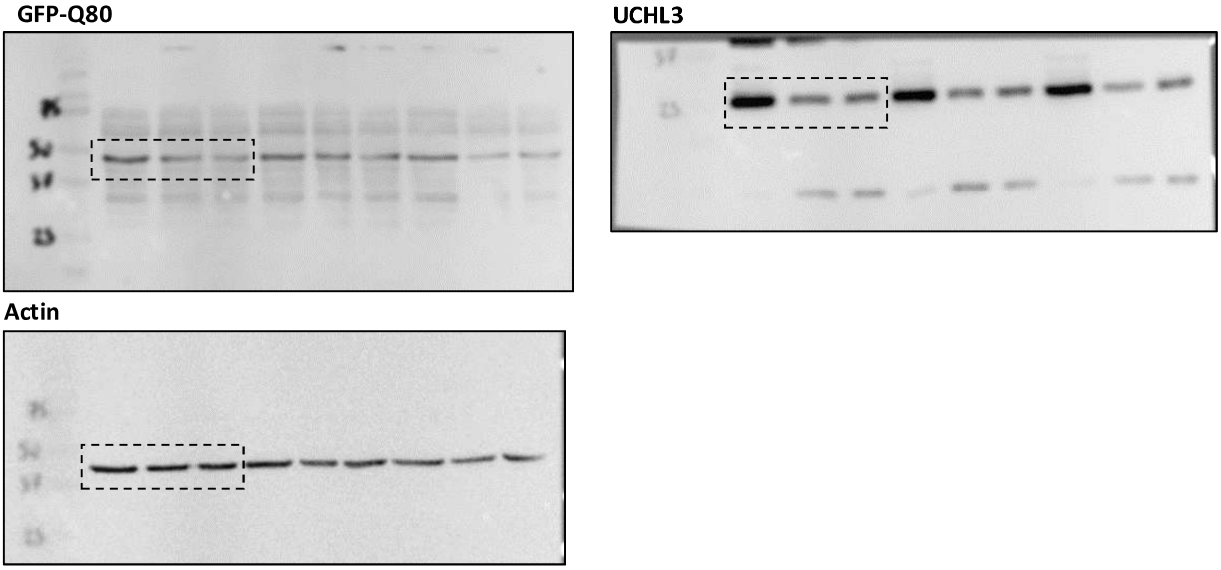

Figure 2C

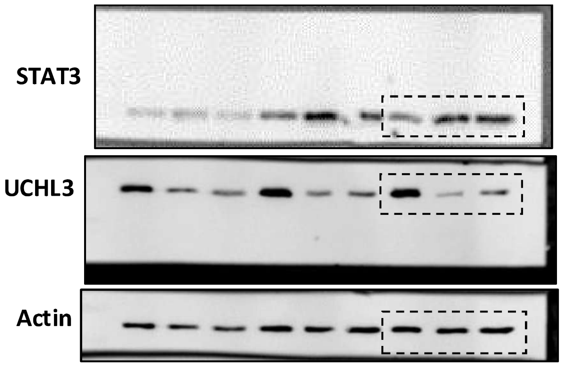

Figure 2D

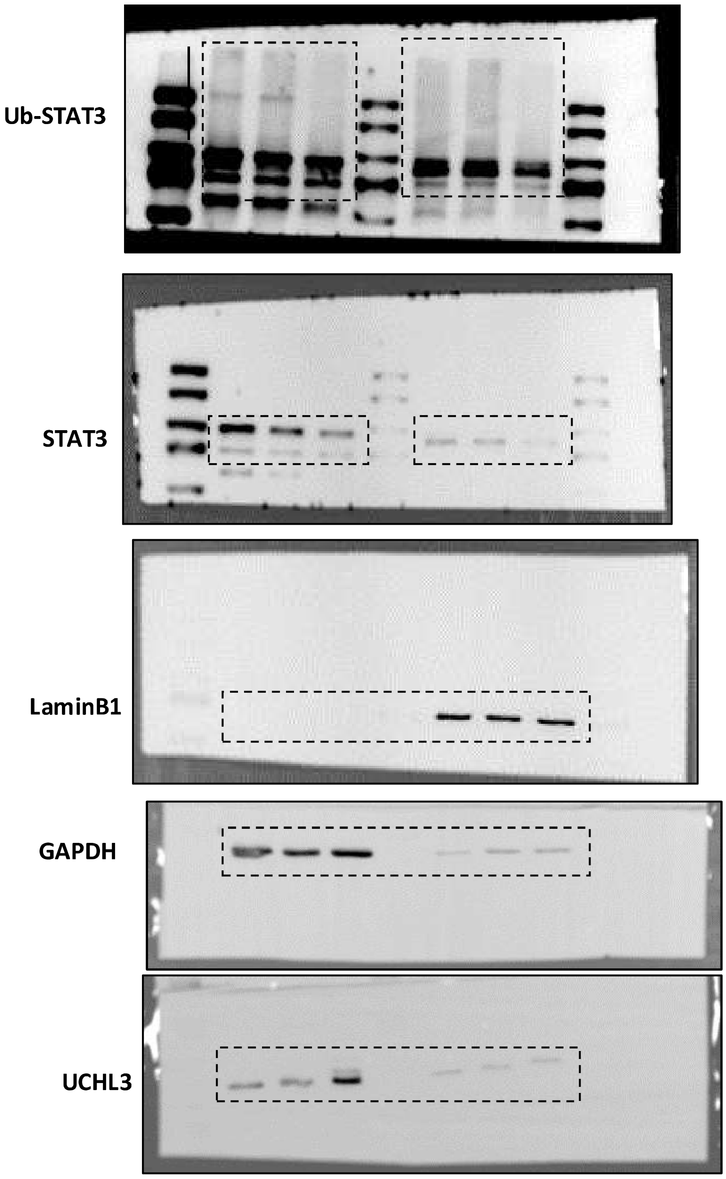

Supplementary Figure 2A

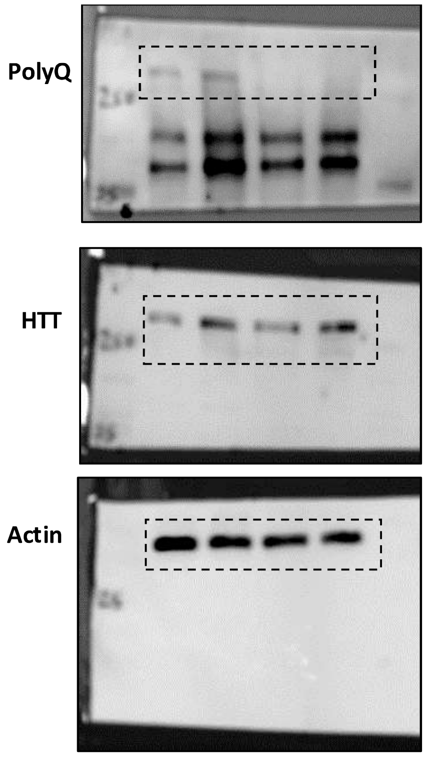

Supplementary Figure 2B

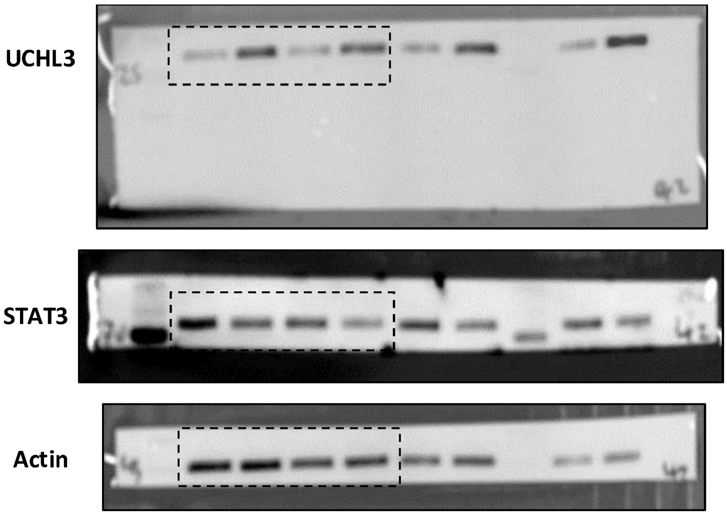

Supplement: awag028_Supplementary_Data [file awag028_supplementary_data.zip › brain-2025-00434-File006.pdf]
